# Incarvine C suppresses proliferation and vasculogenic mimicry of hepatocellular carcinoma cells via targeting ROCK inhibition

**DOI:** 10.1186/s12885-015-1809-5

**Published:** 2015-10-28

**Authors:** Ji-Gang Zhang, Dan-Dan Zhang, Xin Wu, Yu-Zhu Wang, Sheng-Ying Gu, Guan-Hua Zhu, Xiao-Yu Li, Qin Li, Gao-Lin Liu

**Affiliations:** Department of Clinical Pharmacy, Shanghai General Hospital, Shanghai Jiao Tong University School of medicine, No. 100 Haining Road, Shanghai, 200080 P. R. China

**Keywords:** Incarvine C, ROCK, Vasculogenic mimicry, Hepatocellular carcinoma

## Abstract

**Background:**

Studies have described vasculogenic mimicry (VM) as an alternative circulatory system to blood vessels in multiple malignant tumor types, including hepatocellular carcinoma (HCC). In the current study, we aimed to seek novel and more efficient treatment strategies by targeting VM and explore the underlying mechanisms in HCC cells.

**Methods:**

Cell counting kit-8 (CCK-8) assay and colony survival assay were performed to explore the inhibitory effect of incarvine C (IVC) on human cancer cell proliferation. Flow cytometry was performed to analyze the cell cycle distribution after DNA staining and cell apoptosis by the Annexin V-PE and 7-AAD assay. The effect of IVC on Rho-associated, coiled-coil-containing protein kinase (ROCK) was determined by western blotting and stress fiber formation assay. The inhibitory role of IVC on MHCC97H cell VM formation was determined by formation of tubular network structures on Matrigel in vitro, real time-qPCR, confocal microscopy and western blotting techniques.

**Results:**

We explored an anti-metastatic HCC agent, IVC, derived from traditional Chinese medicinal herbs, and found that IVC dose-dependently inhibited the growth of MHCC97H cells. IVC induced MHCC97H cell cycle arrest at G1 transition, which was associated with cyclin-dependent kinase 2 (CDK-2)/cyclin-E1 degradation and p21/p53 up-regulation. In addition, IVC induced apoptotic death of MHCC97H cells. Furthermore, IVC strongly suppressed the phosphorylation of the ROCK substrate myosin phosphatase target subunit-1 (MYPT-1) and ROCK-mediated actin fiber formation. Finally, IVC inhibited cell-dominant tube formation in vitro, which was accompanied with the down-regulation of VM-key factors as detected by real time-qPCR and immunofluorescence.

**Conclusions:**

Taken together, the effective inhibitory effect of IVC on MHCC97H cell proliferation and neovascularization was associated with ROCK inhibition, suggesting that IVC may be a new potential drug candidate for the treatment of HCC.

## Background

Hepatocellular carcinoma (HCC) is the sixth most common malignancy and the third most common cause of cancer-related deaths [1]. Uncontrolled tumor growth, metastasis and the absence of effective therapeutics account for the poor overall survival (OS) of HCC patients. Although surgical resection improves survival, the prognosis for HCC remains unsatisfactory, mainly because of intrahepatic dissemination and extrahepatic metastasis [2]. Angiogenesis is required for metastasis [3, 4], and several mechanisms of tumor vessel formation have been proposed, including vasculogenesis, angiogenesis, intussusceptions, vascular cooption and vasculogenic mimicry (VM). VM describes the *de novo* formation of perfusable, matrix-rich and vasculogenic-like networks by aggressive tumor cells in 3-dimensional (3D) matrices in vitro, which parallels matrix-rich networks in aggressive tumors in patients [5]. Studies have described VM as an alternative circulatory system to blood vessels in multiple malignant tumor types, including HCC [6].

VM, which recapitulates embryonic vasculogenesis [7], was reported to be associated with high tumor grade, short survival, and invasion and metastasis in clinical trials [8–10]. The initial morphologic and molecular characterization of tumor VM cells revealed co-expression of endothelial and tumor markers and formation of functional tubular structures containing plasma and red blood cells, indicating a perfusion pathway for rapidly growing tumors [11]. In addition, the direct exposure of tumor cells lining the inner surface of VM channels to blood flow indicates an escape route for the metastasis process. Considering the diverse nature of vascular perfusion pathways in tumors, it may be prudent to test the efficacy of currently available angiogenesis inhibitors on tumor cell VM in addition to angiogenesis driven by endothelial cells [12].

Rho small GTPase and its serine/threonine kinase downstream effector ROCK play a crucial role in diverse cellular events, including the acquisition of unlimited proliferation potential, survival and evasion from apoptosis, tissue invasion differentiation, gene expression, regulation of cell detachment, cell movement and establishment of metastasis [13, 14]. Recently, growing attention has been paid to the emerging role of the cytoskeleton in the modulation of cell cycle and apoptosis. In some cell types, ROCK is involved in the intracellular signaling that initiates apoptosis, such as Caspase8, Caspase10, and Caspase3 activation [15], or the transcription of proapoptotic proteins, such as Bax [16]. Interestingly, our previous study showed that ROCK was involved in VM formation in an HCC cell line [17], and we hypothesized that unlimited proliferation triggered by ROCK activation may be the key point of regulating tumor cell VM and endothelial cell-driven angiogenesis simultaneously.

Incarvine C (IVC), an ester alkaloid isolated from the traditional Chinese medicinal plant *Incarvillea sinensis* (Bignoniaceae), also known as “Jiaohao (Kakko)” or “Tougucao” [18], has long been used for treating rheumatism and relieving pain in traditional Chinese medicine. However, most alkaloids, originally identified as having anti-inflammatory and anti-viral activities, now are also known to have anti-tumor activities by targeting the apoptosis pathways in cancer [19]. IVC, originally identified as a precursor compound of incarvillateine, has similar activities to morphine [20]. However, the potential effect of IVC on VM and proliferation of highly metastatic HCC cells through ROCK has not been fully studied.

In the current study, with the aim of developing novel and more efficient treatment strategies by targeting VM, we explored the underlying mechanisms of IVC on VM in HCC cells. Our results showed that IVC had a profound inhibitory effect against MHCC97H cell proliferation and migration by promoting MHCC97H cell cycle arrest and apoptotic death. Our findings may serve as strong evidence to suggest that IVC executes a significant inhibitory effect against VM and migration of MHCC97H cells by regulating ROCK, and therefore IVC may prove to be a promising anti-HCC agent.

## Methods

### Chemical and antibodies

Chemicals and antibodies used in this study include Matrigel (BD Biosciences); cell culture media (RPMI 1640, DMEM and MEM), fetal bovine serum (FBS) and antibiotics (Gibco); Y27632 and other chemicals (Sigma-Aldrich); anti-VE-cadherin, MYPT-1 and p-MYPT-1(Thr853) antibodies (Cell Signaling); PE Annexin V Apoptosis Detection Kit and PI/RNase Staining Buffer (BD PharmingenTM). The other reagents were purchased from Abcam Inc. (Cambridge).

### IVC preparation

IVC was extracted as previously described with some modifications [18]. The air-dried powered aerial part (15 kg) of *I. sinensis* was refluxed with 80 % EtOH (30 L). The aq. brownish syrup (6 L) obtained after removing EtOH under reduced pressure was dissolved in 2 % HCl solution and filtered. The filtrate was adjusted to pH 9–10, mixed with NH_4_OH, and extracted with CHCl_3_. The CHCl_3_ extract (68 g) was subjected to column chromatography on silica gel (SiO_2_, 200–300 mesh) with a petroleum ether/AcOEt gradient elution system (100:1 → 5:1) to yield five fractions (1–5). Fraction 5 was purified repeatedly by eluting with CHCl_3_/MeOH (1:2) to afford IVC (6 mg). The IVC solution at a concentration of 20 mg/ml was prepared by dissolving in dimethyl sulfoxide (DMSO). The final DMSO concentration did not exceed 0.5 % throughout the study.

### Cell lines

The HCC cell line MHCC97H was obtained from the Liver Cancer Institute, Zhongshan Hospital of Fudan University (Shanghai, China). HCC cell lines SMMC7703, SMMC7721, HepG2 and Hep3B; prostate carcinoma cells PC3 and LNCap; lung cancer A549 cells; colon cancer HCT116 cells; and cervical cancer Hela cells were from the cell bank of the Chinese Academy of Sciences (Shanghai, China). All cell lines were cultured separately in RPMI 1640 (HUVECs, SMMC7703, SMMC7721, PC3, LNCap, A549), MEM (HepG2 and Hep3B) and DMEM (MHCC97H, HCT116 and Hela), supplemented with 10 % FBS and antibiotics. All cultures were maintained at 37 °C in a humidified atmosphere of 5 % CO_2_.

### Matrigel tube formation assay

Tumor cell formation of the capillary structure in vitro was tested as previously described [17], except that the effect of IVC on tube formation was studied by adding culture medium containing IVC into the wells immediately after cell seeding to a final concentration of 7.5, 15 or 30 μM for 24 h.

### Colony formation assay

MHCC97H cells treated with IVC for 48 h were harvested. A total of 1 × 10^3^ cells per well suspended in 150 μl Mix agar with 1.5 ml DMEM/10 % FBS were plated in a 6-well plate overlying a 1 % agar-DMEM/10 % FBS (1:1) bottom layer. After 3 weeks, colonies were photographed at 4×. The remaining survival large colonies were manually counted.

### Western blot analysis

Cells were treated with different concentrations of IVC or Y27632 for 24 h. Total protein was extracted and electrophoresed using SDS-PAGE. Proteins on the gel were transferred onto PVDF membranes (Merck-Millipore), followed by blocking with 5 % skimmed milk dissolved in TBS containing 1 % Tween-20 (TBST) for 1 h at room temperature. The membrane was incubated with primary antibody at 4 °C overnight, washed with 1 % TBST three times, and incubated with alkaline phosphatase-conjugated secondary antibody for 1 h at room temperature. After washing, the chemiluminescence signal was imaged using ChemiDoc XRS (Bio-Rad) and quantitated using Image J.

### cDNA generation and real-time qPCR

Cells were treated with 7.5, 15, 30 or 60 μM IVC for 16 h. Total RNA was extracted from cells using Trizol (Invitrogen) and verified by electrophoresis. The mRNA was reverse transcribed into cDNA with a reverse transcription kit (Thermo Fisher Scientific). Human GAPDH Endogenous Reference Genes Primers (Order NO.: PHS04) and primer sequences listed in Tables 1 and 2 used for PCR were from Sangon Biotech. SYBR Green I (Amersco) was added into the reaction mixture. Real-time qPCR was performed on an MJ Opticon 2 thermal cycler (MJ Research Inc.) following the manufacturer’s instruction.

**Table 1:** Sequences of primers

Gene	Forward Primer (5’-3’)	Reverse Primer (5’-3’)	Size
CDK-2	TACTTCTATGCCTGATTACA	TGGGGTACTGGCTTGGTCAC	199
CDK-4	GCTACCAGATGGCACTTACACC	GCAAAGATACAGCCAACACTCC	118
CDK-6	GCCCACTGAAACCATAAAGGA	TACCACAGCGTGACGACCA	204
Cynlin-A1	TGTCACCGTTCCTCCTTGG	GGGCATCTTCACGCTCTATTT	125
Cynlin-B1	GCTCTTGGGGACATTGGTAAC	CAAAATAGGCTCAGGCGAAA	234
Cynlin-C1	TTGATTGCTGCTGCTACTTCTG	CGTTCTGTAGGTATCATTCACT	237
Cynlin-D1	GGAACAGAAGTGCGAGGAGG	GGATGGAGTTGTCGGTGTAGAT	191
Cynlin-E1	CCTGGATGTTGACTGCCTTGA	TGTCGCACCACTGATACCCT	113
p21	GACACCACTGGAGGGTGACT	CACATGGTCTTCCTCTGCTG	166
p27	TAGAGGGCAAGTACGAGTGG	CAGGTCGCTTCCTTATTCC	258
P53	GTTCCGAGAGCTGAATGAGG	TGAGTCAGGCCCTTCTGTCT	157

All the sequences are based on the published data on the National Center for Biotechnology Information followed by the accession number

**Table 2:** Sequences of primers

Gene	Forward Primer (5’-3’)	Reverse Primer (5’-3’)	Size
VE-cadherin	CATTTGTCGTGCCTGAAGAC	ATGGTGAAAGCGTCCTGGTA	133
EphA2	CCCGGAGGACGTTTACTTCT	GGATGGATGGATCTCGGTAG	124
PI3K	CGTTTCTGCTTTGGGACAAC	CCTGATGATGGTCGTGGAG	100
MMP14	TATGGCTTCTGCCCTGAGAC	GGGTTTCTTCTGCCCACTT	120
MMP2	TATGGCTTCTGCCCTGAGAC	CACACCACATCTTTCCGTCA	142
MMP9	CAGTCCACCCTTGTGCTCTT	ACTCTCCACGCATCTCTGC	118
LAMC2	ACATTCCTGCCTCAGACCAC	TCCCTTGTCAGTTGCTCCAT	121

All the sequences are based on the published data on the National Center for Biotechnology Information followed by the accession number

### Immunostaining

After fixation for 20 min in 4 % paraformaldehyde at 37 °C, cells were permeabilized for 15 min in 0.5 % Triton-X and subsequently blocked in 5 % goat serum for 1 h. After incubation with the appropriate primary (overnight incubation at 4 °C) and secondary (2 h at 37 °C) antibodies, the cells were imaged using a confocal laser scanning microscope (Leica TCS SP8).

### Cell morphology assay

MHCC97H cells were plated at 8 × 10^3^ cells per well in an 8-chamber slide in serum-free medium (serum starvation) for 24 h. After serum starvation, cells were treated with vehicle, Y27632 or IVC for 1 h. After treatment, cells were fixed with 4 % paraformaldehyde, permeabilized using 0.1 % Triton X-100 and stained with phalloidin (Sigma, USA) and DAPI (blue). Cells were imaged using a confocal laser scanning microscope (Leica TCS SP8).

### Cell migration assay

Cell migration was evaluated using an in vitro wound healing assay. In brief, MHCC97H cells containing IVC at a final concentration of 7.5, 15 or 30 μM were seeded to a six-well plate and incubated for 8 h to allow the formation of a cell monolayer. Cells were scratched with the tip of a 200 μl pipette and then incubated at 37 °C in a humidified atmosphere of 5 % CO_2_ for 48 h. To observe the wound healing, each well was observed at 0, 24 and 48 h after scratching.

### Matrigel invasion assay

Invasion was assayed in Transwell cell culture chambers (Corning Costar) attached with a membrane filter (8.0 μm pore size; Nucleopore). Briefly, the inserts in the membrane filter were coated with Matrigel on the upper surface. MHCC97H cells were adjusted to a density of 1 × 10^6^ cells/ml in serum-free DMEM high glucose culture medium. Cell suspension (200 μl) was seeded into the upper surface, while the lower chambers were filled with 500 μl DMEM medium with 20 % FBS. After culture at 37 °C in a humidified atmosphere of 5 % CO_2_ for 48 h, the cells that invaded the Matrigel matrix and adhered to the bottom surface of the membrane were fixed with methanol and stained with 0.5 % crystal violet. The number of invading cells was counted using an inverted light microscope (Nikon, Japan). Assays were performed in duplicate wells.

### Cell apoptosis, cell cycle and cell viability assessment

Apoptosis and cell cycle of MHCC97H cells exposed to IVC (3.75, 7.5, 15, 30 or 60 μM) for 24 h were examined by flow cytometry (BD Accuri C6). For the apoptosis assay, cells were stained with PE Annexin V and 7-amino-actinomycin (7-AAD) following the manufacturer’s instructions to detect early apoptotic cells (PE Annexin V+/7-AAD- events) and late apoptotic cells (PE Annexin V+/7-AAD+ events). For the cell cycle assay, cells were fixed with 70 % ethanol overnight at 4 °C, washed with PBS, re-suspended in 500 μl PBS with 100 μg/ml RNase and incubated for 30 min at 37 °C. Next, 2.5 μl PI solution (10 mg/ml) was added and cell cycle was analyzed. For cell viability assays, MHCC97H cells were trypsinized and seeded at 1 × 10^4^ cells/well in a 96-well plate. After 24 h, various concentrations of IVC were added, followed by incubation for another 48 h. Next, 10 μl CCK8 (Dojindo) solution was added to each well and the plate was incubated for additional 2 h. Absorbance readings at 490 nm were obtained using a spectrophotometer (Thermo Varioskan).

### Statistical analysis

The data were expressed as mean ± standard errors (S.E.) and examined for their statistical significance of difference with ANOVA and the Student’s *t*-test. *P*-values of less than 0.05 were considered statistically significant.

## Results

### IVC inhibits the proliferation of human cancer cells

**Fig. 1:**
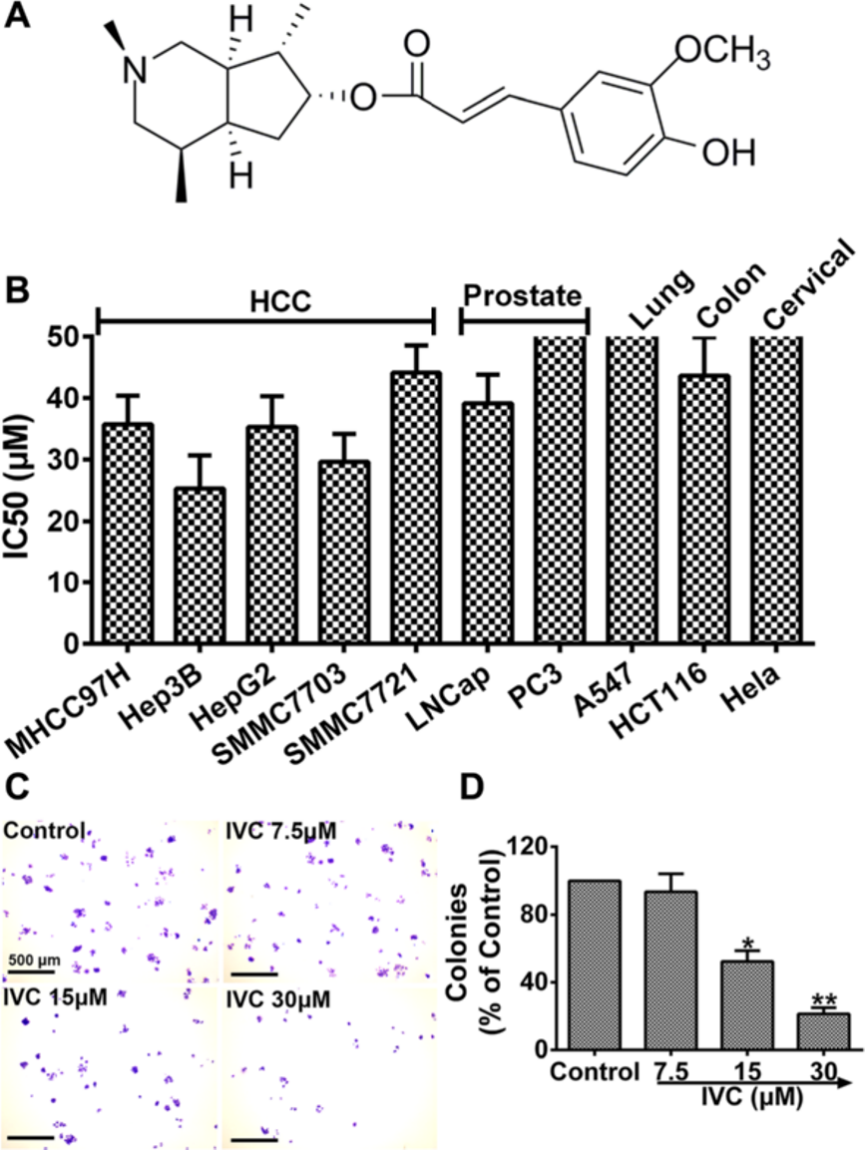
Inhibitory effects of IVC on the proliferation of different human cancer cell lines. **a** Structure of IVC. **b** Various cancer cell lines were incubated with IVC for 48 h, and cell growth was measured by CCK-8 assay. **c** and **d** Colony formation assay was performed after MHCC97H cells were treated with IVC at indicated doses, and the number of colonies was manually counted. Original magnification was 40×, scale bars represent 500 μm

Uncontrolled proliferation and robust angiogenesis (i.e. VM) contribute to the growth and metastasis of cancers. Thus, CCK-8 assay was first used to determine the effect of IVC on the proliferation of human cancer cells. The chemical structure of IVC is shown in Fig. 1a. IVC inhibited the proliferation of different cancer cells in a dose-dependent manner (data not shown). As shown in Fig. 1b, IVC inhibited the proliferation of MHCC97H cells in a dose-dependent manner with an IC_50_ value 35.7 ± 4.7 μM. These cells had been shown to have capacity of VM formation in our previous study [17]. We next performed colonial survival assay under more stringent conditions to further explore the inhibitory effect of IVC on MHCC97H cell proliferation. The results showed that the number of remaining survival colonies in the IVC-treated groups at 15 and 30 μM was significantly lower than that of the control group (*P < 0.05 and **P < 0.01; Fig. 1c and d). Together these results indicate that IVC effectively inhibited MHCC97H cell proliferation in vitro. We thus used MHCC97H cells as a model to study the anti-VM potency and mechanism of IVC in subsequent analyses.

### IVC affects cell cycle progression of MHCC97H cells

**Fig. 2:**
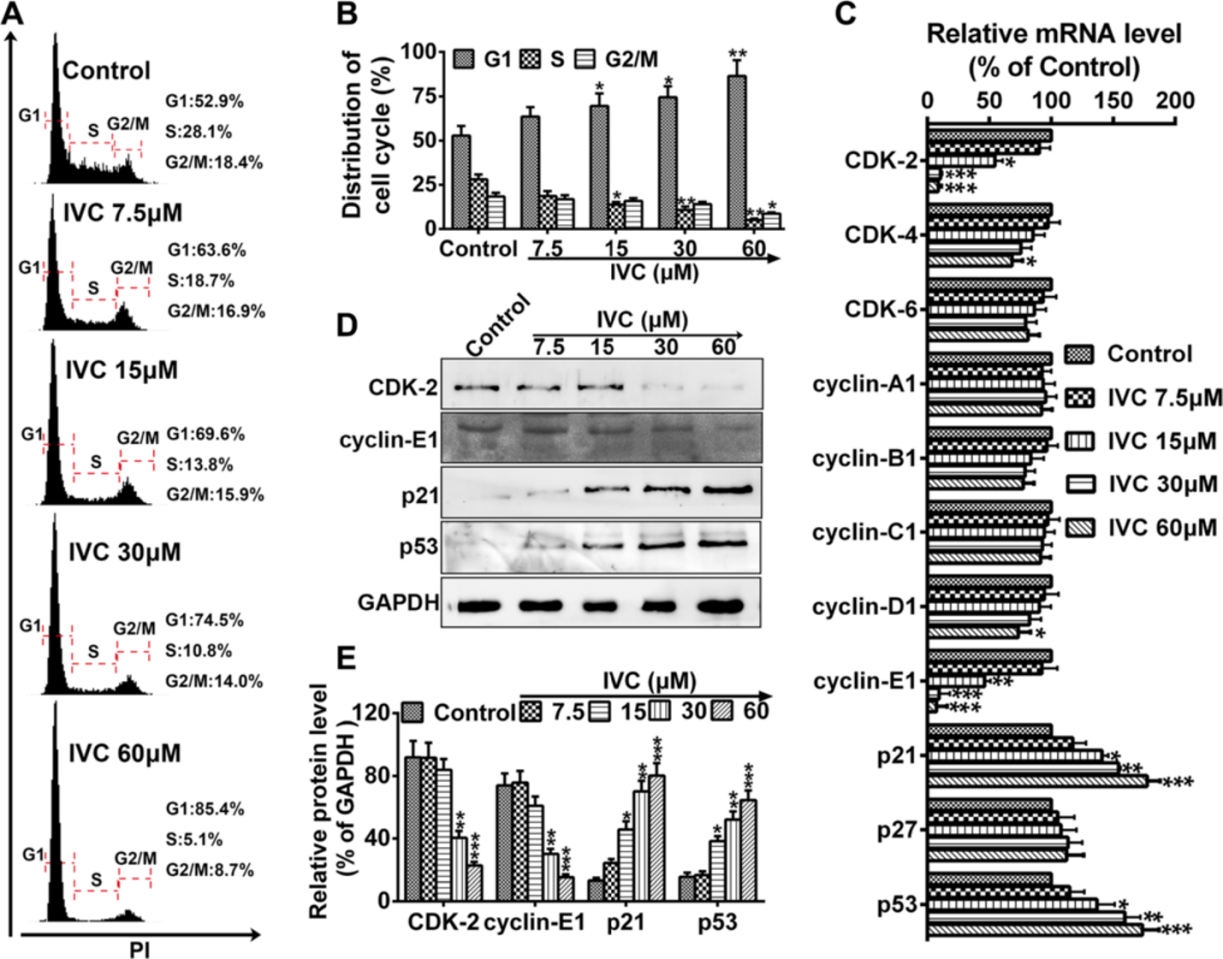
IVC affects cell cycle progression. **a** MHCC97H cells were incubated with IVC at indicated doses for 24 h, and the DNA content of propidium iodide-stained cells was analyzed by flow cytometry. **b** Cell cycle distribution was analyzed. **c** mRNAs of cell cycle regulatory genes were detected by real time-qPCR assay. **d** Protein expressions of CDK-2, cyclin-E1, p21, and p53 were detected by western blot. **e** The relative protein level in each condition was quantitated using Image J and represented as a line chart corresponding to pixels detected. Data are expressed as mean ± S.E. (*n* = 3). *P < 0.05, **P < 0.01 and ***P < 0.001 vs. the control

To investigate the mechanism of the inhibitory effect of IVC on MHCC97H cell growth, flow cytometry was performed to analyze the cell cycle distribution after DNA staining. IVC treatment at doses of 15, 30 and 60 μM significantly increased the MHCC97H cell population at the G1 phase (*P < 0.05 and **P < 0.01, vs. control; G0 phase not shown, *P* > 0.05 vs. control; Fig. 2a and b). We next performed real time-qPCR analyses of key cell cycle regulatory genes (primers are shown in Table 1). Real time-qPCR results showed that the mRNA expressions of CDK-2 and cyclin-E1 were dramatically down-regulated after treatment of cells with IVC (15, 30 and 60 μM), and CDK-4 and cyclin-D1 were slightly decreased after IVC treatment at doses of at least 60 μM, while p21 and p53 mRNA levels were markedly increased (*P < 0.05, **P < 0.01 and ***P < 0.001 vs. control; Fig. 2c). However, the expression levels of several other cell cycle regulators including CDK-6, cyclin-B1 and p27 were not significantly affected (*P* > 0.05 vs. control; Fig. 2c). Furthermore, western blot confirmed that the protein level of CDK-2 and cyclin-E1 was markedly decreased after 30 and 60 μM IVC treatment, while p21 and p53 protein expressions were significantly upregulated after 15, 30 and 60 μM IVC treatment (*P < 0.05, **P < 0.01 and ***P < 0.001 vs. control; Fig. 2d and e). Collectively, these results suggest that IVC suppressed MHCC97H cell proliferation by inducing cell cycle arrest at the G1 phase via decreasing the expression of CDK-2 and cyclinE1 and enhancing the expression of p21 and p53 genes.

### IVC induces apoptotic death of MHCC97H cells

**Fig. 3:**
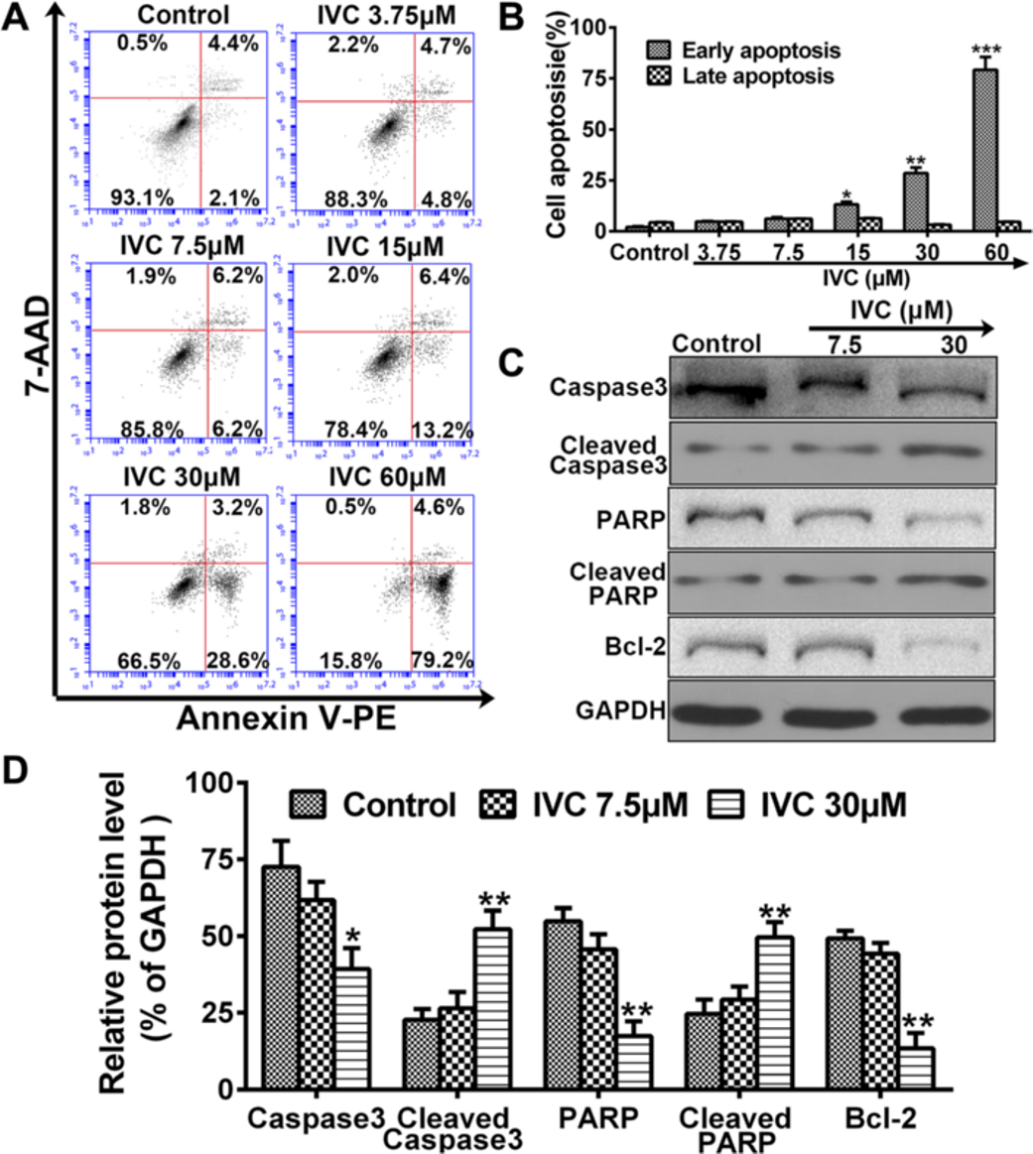
IVC induces apoptotic death. **a** MHCC97H cells were incubated with IVC at indicated doses for 24 h, and Annexin V-PE and 7-AAD stained cells were sorted by flow cytometry. **b** Distribution of cell apoptosis was analyzed. **c** Protein expressions of Caspase3, Cleaved Caspase3, PARP and Bcl-2 were detected by western blot. **d** The relative protein level in each condition was quantitated using Image J. Experiments were repeated three times, and similar results were obtained. Data are expressed as mean ± S.E. *P < 0.05, **P < 0.01 and ***P < 0.001 vs. the control

To test whether IVC-mediated growth inhibition was due to the induction of cell apoptosis, the Annexin V-PE and 7-AAD assay was used to examine apoptosis in MHCC97H cells treated with various concentrations of IVC. As shown in Fig. 3a and b, the population of early apoptotic (PE Annexin V+/7-AAD- events) MHCC97H cells was increased significantly after highdose (>7.5 μM) IVC treatment (*P < 0.05, **P < 0.01 and ***P < 0.001 vs. control). Consistent with these results, poly (ADP-ribose) polymerase (PARP), Caspase3 and Bcl-2 were down-regulated after IVC treatment, while cleaved Caspase3 and cleaved PARP were up-regulated (*P < 0.05 and **P < 0.01 vs. control; Fig. 3c and d). Together these results suggest that apoptotic cell death might contribute to IVC-induced anti-proliferation effect in MHCC97H cells.

### IVC mediates biological effect via inhibition of ROCK

**Fig. 4:**
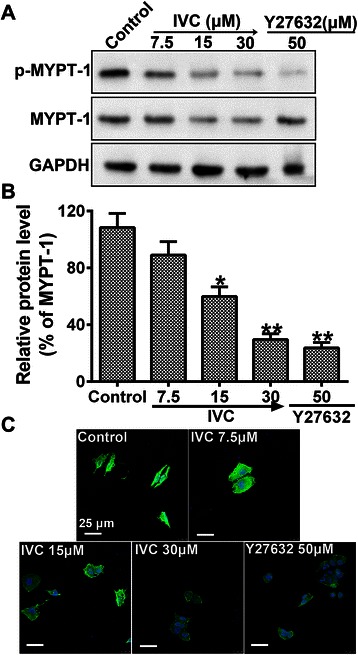
IVC as a mediator of ROCK. **a** MHCC97H cells were treated with increasing concentrations of IVC for 24 h and p-MYPT-1 was examined by western blot. ROCK inhibitor Y27632 was used as the positive control. **b** The relative protein level in each condition was quantitated using Image J. **c** IVC inhibits the formation of stress fibers. Starved MHCC97H cells were treated with either vehicle, Y27632 or IVC for 1 h and then fixed and stained with phalloidin (green) and DAPI (blue). Original magnification was 400×, scale bars represent 25 μm. Data are representative of three independent experiments. Data are expressed as mean ± S.E. *P < 0.05 and **P < 0.01 vs. the control

To investigate the effect of IVC on ROCK, MHCC97H cells were treated with indicated doses of IVC and analyzed by western blotting to evaluate phosphorylated levels of the ROCK substrate MYPT1 (p-MYPT-1) and total level of MYPT-1. Y27632 (ROCK inhibitor) was included as a positive control. Figure 4a shows that IVC treatment decreased the level of p-MYPT-1 in a concentration-dependent manner. Furthermore, IVC at 30 μM was more potent in decreasing p-MYPT-1 at all concentrations, which was similar to the case with 50 μM Y27632 as shown in Fig. 4b.

Stress fiber formation is a cytoskeleton re-organization process mediated by the activation of the Rho/ROCK pathway. To determine the effects of IVC on these morphological changes, MHCC97H cells were starved and treated with IVC, Y27632 or vehicle, and then stained with phalloidin as described in Methods. Figure 4c shows that IVC dose-dependently inhibited stress fiber formation, which is known to be critical to cell motility, suggesting that this regulator may interfere with the ability of cancer cells to migrate and invade, or even VM.

### IVC suppresses HCC cell motility

**Fig. 5:**
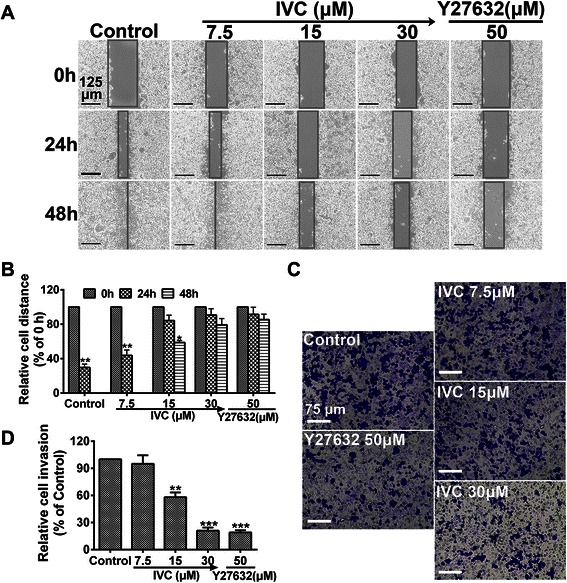
Inhibitory effect of IVC on MHCC97H cell motility. **a** Cells were plated, incubated for 8 h, scratched and treated with vehicle, IVC or Y27632. Representative images of scratch wounds formed at 0, 24 and 48 h after wounding. Original magnification was 100×, scale bars represent 125 μm. **c** MHCC97H cells were treated with vehicle, IVC or Y27632, plated in Corning Transwell inserts coated with Matrigel, and allowed to invade for 48 h. Original magnification was 200×, scale bars represent 75 μm. **b** and **d** Data were normalized to untreated cells and the relative migration or invasion is expressed as mean ± S.E. of triplicate experiments. *P < 0.05, **P < 0.01 and ***P < 0.001 vs. the control

VM is believed to be associated with cell migration and invasion. To explore the function of IVC in the migration ability of MHCC97H cells, IVC (7.5, 15 and 30 μM) was used in wound-healing and invasion assays, using Y27632 (50 μM) as positive control. In untreated MHCC97H cells, cell migration toward the wounded area was observed and cells were grown to near confluence within 24 h, while addition of IVC (30 μM) to cells blocked their migration within 48 h (*P* > 0.05 vs. 0 h; Fig. 5a and b). These results indicate that cultured cells treated with IVC failed to migrate. Knowing that the ability of cancer cells to metastasize depends on their ability to invade, we next investigated the ability of IVC to inhibit cell invasion. Figure 5c showed that cell invasion decreased by about 44 % and 73 % after incubation with 15 μM and 30 μM IVC, respectively, compared with control cells (**P < 0.01 and ***P < 0.001; Fig. 5d). No significant difference in the wound-healing rate and invasion activity was observed between positive control cells and cells treated with IVC (30 μM). These results suggest that IVC inhibited cell motility and VM formation in a dose-dependent manner.

### IVC suppresses VM formation in MHCC97H cells

**Fig. 6:**
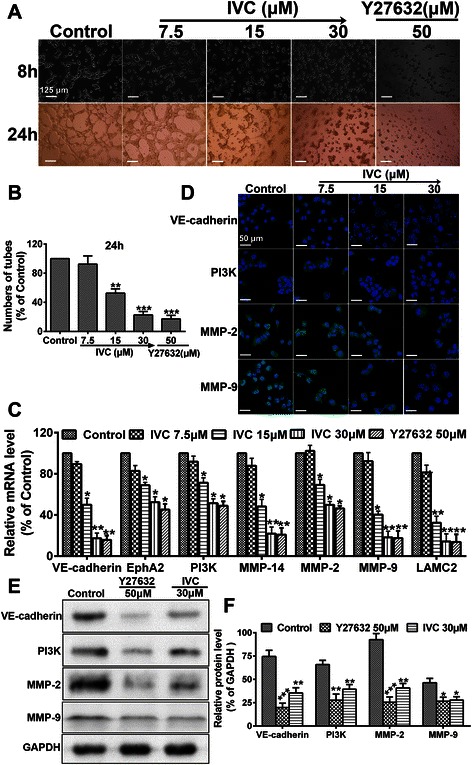
The impact of IVC on VM formation. **a** VM formation of MHCC97H cells cultured in a Matrigel-coated 24-well plate with culture medium containing IVC (7.5, 15 or 30 μM) or Y27632 (50 μm) for 8 h or 24 h. Photographs were taken at the indicated time points. Original magnification was 100×, scale bars represent 125 μm. **b** Quantitative analysis of the mean number of tube-like structures formed from six randomly chosen areas in 3D cultures. **c** Real-time qPCR analysis was used to determine changes in gene expression. Relative IVC-induced change in gene expression compared with GAPDH is expressed as fold change calculated by 2^-ΔΔ^Cp method. Y27632 (50 μM) was used as positive control. **d** Representative confocal images (*n* = 3, five pictures per condition) of control and IVC (7.5, 15 or 30 μM) with 24 h incubation after immunostaining for VE-cadherin, PI3K, MMP-2 or MMP-9 (green) and DAPI (blue). Scale bars represent 50 μm. **e** Western blot assay was performed to evaluate the effect of IVC or Y27632 on VM markers, using GAPDH as an internal control for protein loading. **f** Relative protein level was quantitated using Image J. Data are expressed as mean ± S.E. from three independent experiments, with significant differences from control designated as *P < 0.05, **P < 0.01 and ***P < 0.001

To detect whether IVC had an inhibitory effect on MHCC97H cell-mediated neovascularization in vitro, we observed the morphology of cell seizure activity in culture medium containing IVC, using Y27632 (50 μM) as the positive control. MHCC97H cells were seeded onto Matrigel, and the formation of VM structures was monitored and photographed after 8 and 24 h (Fig. 6a). MHCC97H cells without treatment gradually formed characteristic tubular structures within 8 h and were completed within 24 h. IVC disrupted the formation of tubule-like structures in a dose-dependent manner (**P < 0.01 and ***P < 0.001 vs. control; Fig. 6b). Compared with the control, treatment with 30 μM IVC reduced VM formation by almost 85 %.

To further examine the mechanism underlying the IVC-mediated anti-VM effect, VM-associated genes, including VE-cadherin, EphA2, PI3K, MMP-14, MMP-2, MMP-9 and LAMC2, were evaluated using real-time qPCR (primers shown in Table 2). Similar to the 50 μM Y27632 positive control, mRNA levels were significantly down-regulated by IVC (15 and 30 μM) compared with the controls (*P < 0.05 and **P < 0.01; Fig. 6c). Furthermore, immunofluorescence (Fig. 6d) and western blot analyses (Fig. 6e and f) confirmed that VE-cadherin, PI3K, MMP-2 and MMP-9 proteins were down-regulated by IVC in MHCC97H cells. These results demonstrate that IVC had the potential to exogenously affect VM activity of MHCC97H cells, and strongly imply that IVC may suppress VM development through the Rho/ROCK pathway.

## Discussion

Gene-expression analysis of human HCC has led to the successful molecular classification of HCC into six robust subgroups of cancers (G1–G6) associated with clinical and genetic characteristics [21]. Despite the recent advances in treatment, the mortality rate of HCC remains high, and therefore new effective anti-HCC drugs are urgently needed. Better understanding about the molecular mechanism of HCC invasiveness is essential for the development of effective treatment of HCC. It is generally recognized that the high metastatic ability of HCC cells is the critical reason for the fatalities associated with HCC [22]. The growth and metastasis of HCC cells depend on an effective microcirculation. Effective microcirculation in aggressive tumors consists of vasculogenesis, angiogenesis and VM causing resistance to conventional anti-angiogenic medicaments, and thus many researchers have been seeking to develop new angiogenic and VM inhibitors from cleaved proteins, monoclonal antibodies, synthesized small molecules and natural products [23, 24]. These new anti-vascular therapeutic agents should be able to target both angiogenesis and VM, anti-VM therapy for tumor VM [25]. Although IVC was originally identified as a precursor compound of incarvillateine, details about the pharmacological mechanism underlying the anti-tumor activity of IVC have not been clarified. The results of the present study demonstrated that IVC exhibited remarkable inhibitory effect on several types of cancer cells, including MHCC97H cells. Importantly, IVC dose-dependently inhibited the proliferation of MHCC97H cells, which are known to have the capacity of VM formation [17], suggesting that IVC is a potential new anti-HCC drug candidate.

Deregulation of the cell cycle leads to robust tumor cell proliferation, which is an important hallmark of cancer [26]. Progression through the S-phase must be strictly controlled so that cells undergo only a single round of chromosomal DNA replication. Our cell cycle analyses demonstrated that IVC caused the accumulation of MHCC97H cells at G1 phase and only CDK-2 and cyclin-E1 were down-regulated after IVC treatment. Previous studies reported that the CDK-2/cyclin-E1 complex plays a crucial role during the transition from G1- into S-phase [27]. CDK inhibitors including p21 and p27 and the tumor suppressor p53 also play a key role in regulating cell cycle progression through suppressing CDK-2 activity and G1/S phase transition [28, 29]. Our real time-qPCR and western blot results showed that p21 and p53 were up-regulated after IVC treatment, without any changes on the other CDKs/cyclins, suggesting that IVC induced MHCC97H cell cycle arrest at G1 phase probably via upregulating p21 and p53. We also found that 15 μM IVC treatment induced significant cell apoptosis, and this IVC-induced apoptosis may be associated with PARP and Caspase3 degradation, which is consistent with other studies [30]. These results suggest that IVC may be a potential antiproliferation agent for HCC.

Accumulating evidence indicates that Rho and the downstream target ROCK plays an important role in oncogenesis. ROCK promotes actin filament stabilization and the generation of actin-myosin contractility. Y27632 or fasudil, inhibitors of ROCK, caused loss of actin stress fibers and focal adhesion complexes [31]. We found that treatment of MHCC97H cells with IVC decreased the level of p-MYPT-1 and distributed stress fiber formation in a concentration-dependent manner similar to the case with Y27632 (Fig. 4), indicating that IVC is a potential mediator of Rho/ROCK. Given the volume of the literature supporting a role for Rho/ROCK in controlling cell movement, it is not surprising to find that inhibition of IVC blocked MHCC97H migration and invasion, which is similar to the finding of our previous study [17]. The inhibitory effects of IVC with different concentrations on cell migration and invasion and ROCK activity were below IC_50_ value of 35.7 ± 4.7 μM in order to avoid potential toxicity. Thus, IVC could effectively exert a regulated impact on cell migration and invasion, but are hardly associated with cytotoxicity of the compound.

Rho GTPases contribute to multiple cellular processes that could affect cancer progression, including cytoskeletal dynamics, transcriptional regulation, vesicle trafficking, apoptosis, cell cycle progression and migration [32]. ROCK activity is necessary for progression from G1 to S-phase by controlling the expression of cyclins, CDKs, and numerous other cell cycle regulators [33]. Furthermore, ROCK activity has been shown to promote CDK-2 and cyclin-E1 translocation into the nucleus [34], suggesting that inhibition of ROCK signaling may lead to cell cycle arrest in G1 phase. Moreover, ROCK inhibition has been shown to increase phosphorylation of p53 [35, 36]. Additionally, ROCK proteins are essential for multiple aspects of both the intrinsic and extrinsic apoptotic processes, including regulation of cytoskeletal-mediated cell contraction and membrane blebbing, nuclear membrane disintegration, modulation of Bcl-2 family member and caspase expression/activation and phagocytosis of fragmented apoptotic bodies [34]. This finding could serve as an explanation why IVC not only mediates G1 arrest but also induces apoptosis. Together our results suggest that IVC mediates the proliferation of human cancer cells via downregulating the Rho/ROCK pathway.

However, our previous finding that ROCK inhibitor: Y27632 at the concentrations up to 50 μM does not essential for survival and proliferation of MHCC97H cells [17], which is different to the results of IVC, suggests Y27632 and IVC have distinct effects on cell proliferation and apoptosis. In respect of these differences, we found that long-term treatment with fasudil improved pulmonary vascular remodeling with suppression of vascular smooth muscle cells (VSMC) proliferation and enhancing of VSMC apoptosis [37], which is similar to the result of IVC but is different to Y27632 [17], indicating that each compound has various functions with respective or unique mechanism. Interestingly, Y27632 has been reported to have opposite effects on cell motility. Koga et al. [38] reported that Y27632 promoted human trabecular meshwork cells in adhesion, contraction and motility, which was inconsistent with our or other reports [17, 39, 40], implying that same compound has a wide variety of effects on different cell line. Moreover, growing number of ROCK inhibitors have been reported, however, their pharmacological effects varied in different diseases [41].

Besides, it is well known that ROCK have two subtypes: ROCK1 and ROCK2. However, Y27632 targets the highly conserved kinase domain of ROCK, which does not distinguish between ROCK1 and ROCK2 isoforms. Inhibition of ROCK1 resulted in a decreased proliferation, whereas inhibition of ROCK2 had the opposite effect, significantly enhancing proliferation relative to the control cells and regulating cyclin D1 [42–44] to mediate the canonical Wnt/TCF pathways involving β-catenin. Therefore, the relative contribution of these kinases to the effects of Y27632 treatment differ in different cell types and cellular processes. This is an important consideration in developing specific therapeutics tailored to distinct cellular responses and diseases. Taken together, our manuscript showed that Y27632 and IVC have distinct effects on cell proliferation and apoptosis, further investigation will be performed to explain what’s different.

Our previous study reported that selective blockage of ROCK with Y27632 reduced the formation of tubule-like structures in a dose-dependent manner [17]. Therefore, we further investigated the anti-VM activity of IVC. Similarly, here we found that IVC reduced VM in a dose-dependent manner compared with Y27632. The key signaling pathway of VM formation is the colocalization of VE-cadherin and activation of PI3K by EphA2, which subsequently induce the expression and activation of MMP-14, which subsequently triggers MMP-2 activation. MMP-14 and MMP-2 promote the cleavage of LAMC2 to the pro-migratory γ2′ and γ2 × fragments. Our investigation showed that VM-associated genes were down-regulated, suggesting that IVC could attenuate the characteristics associated with tumor cell plasticity essential for VM. To prove IVC as a potential anti-VM candidate, we will continue to perform animal experiments to examine tumor size, invasion and VM-associated genes in nude mice.

## Conclusions

Our data presented here demonstrate that IVC, as a mediator of ROCK, possesses anti-HCC ability by inducing cell cycle arrest and cell apoptosis as well as suppressing tumor cell migration and VM in vitro. Thus, IVC may be a potential anti-VM candidate for anti-HCC therapy.

## Abbreviations

HCC: Hepatocellular carcinoma
IVC: Incarvine C
VM: Vasculogenic mimicry
CDK-2: Cyclin-dependent kinase 2
MYPT-1: Myosin phosphatase target subunit-1
EphA2: Erythropoietin-producing hepatocellular carcinoma-A2
PI3K: Phosphoinositide 3-kinase
MMP-14: Matrix metalloproteinase-14
LAMC2: Laminin 5γ2-chain
OS: Overall survival
3D: 3-dimensional
DMSO: Dimethyl sulfoxide
FBS: Fetal bovine serum
